# Obesogenic eating behaviors mediate the relationships between psychological problems and BMI in children

**DOI:** 10.1002/oby.21823

**Published:** 2017-03-29

**Authors:** Kimberley M. Mallan, Lynne A. Daniels, Jan M. Nicholson

**Affiliations:** ^1^ School of Psychology Australian Catholic UniversityBrisbaneAustralia; ^2^ School of Exercise and Nutrition SciencesQueensland University of TechnologyBrisbaneAustralia; ^3^ Institute of Health and Biomedical InnovationQueensland University of TechnologyBrisbaneAustralia; ^4^ Judith Lumley Centre, La Trobe UniversityMelbourneAustralia; ^5^ School of Early ChildhoodQueensland University of TechnologyBrisbaneAustralia

## Abstract

**Objective:**

To examine the association between psychological problems and weight status in children aged 3.5 to 4 years and test whether obesogenic eating behaviors mediate this relationship.

**Methods:**

This study is a cross‐sectional secondary analysis of data from first‐time mothers (*N* = 194) in the control arm of the NOURISH randomized controlled trial. At child age 3.5 to 4 years, maternal‐reported child eating behaviors and psychological problems were collected via valid tools, and child weight and height data were collected by trained study staff. Pearson's correlations and linear regressions examined associations between eating behaviors, psychological problems, and BMI *z* score. Multiple mediation models were tested by assessing indirect effects of psychological problems on BMI *z* score via obesogenic eating behaviors.

**Results:**

Peer problems were associated with both higher food responsiveness and emotional overeating and directly with higher BMI *z* score. This relationship was partially mediated by emotional overeating. Both emotional overeating and food responsiveness fully mediated the association between emotional problems and BMI *z* score, and food responsiveness fully mediated the association between conduct problems and BMI *z* score.

**Conclusions:**

The findings suggest that children with psychological problems may also display obesogenic eating behaviors, which may result in higher BMI. This needs to be considered in the clinical management of both pediatric overweight/obesity and psychological problems.

## Introduction

Childhood overweight/obesity and emotional and behavioral problems (i.e., psychological problems) are common public health issues that appear to be interrelated. Children with overweight and obesity are more likely to experience a range of psychological problems compared to their healthy‐weight peers 1, 2, 3, 4. In adolescent and adult populations, high BMI negatively impacts on a range of wellbeing measures 5 and increases the risk for future psychological problems 1, 6, while psychological problems prospectively predict higher BMI 7, 8, 9. In young children, support for the latter relationship (psychological problems to overweight) is less robust 1. Improving our understanding of how BMI and psychological problems may be related during childhood is critical to designing both preventive and treatment interventions.

The association between BMI and psychological problems appears to emerge early in life. Cross‐sectional and longitudinal findings from the UK's Millennium Cohort Study (*N* = 11,202) examined associations between weight status (nonoverweight, overweight, obesity) and psychological problems at 3 and 5 years for boys and girls separately 10, using the parent‐reported Strengths and Difficulties Questionnaire (SDQ), a widely used, relatively brief measure that has been shown to have good reliability and validity 11, 12. The SDQ assesses four problem domains divided into internalizing problems (emotional symptoms and peer problems) and externalizing problems (conduct problems and hyperactivity), as well as a prosocial behavior domain. Associations between obesity and psychological problems emerged as early as 3 years of age, with boys with obesity having more conduct problems than healthy‐weight boys. At 5 years of age, boys with obesity had more conduct problems, hyperactivity, and peer problems, and girls with obesity had more peer problems compared to their nonoverweight counterparts 10.

Other large population‐based studies also have found evidence of an association between psychological problems and higher BMI in young children. In the Longitudinal Study of Australian Children (LSAC; *N* = 4,983), associations at age 4 to 5 years between weight status and psychological problems (assessed using the SDQ 11 completed by parents and teachers) were examined for boys and girls separately 13. While children with overweight/obesity had more problems than their nonoverweight peers, differences were small and many associations became nonsignificant after adjusting for a range of sociodemographic covariates. The most robust associations were more peer problems (parent‐reported) for girls and more conduct problems (teacher report) and total difficulties (teacher report; sum of four problem scales) for boys across the nonoverweight, overweight, and obesity groups. A follow‐up of these children at age 8 to 9 years (*N* = 3,363) found that BMI *z* score at age 4 to 5 was prospectively associated with increased odds of scoring in the abnormal range for peer problems (parent and teacher report), emotional symptoms (teacher report), and total difficulties (teacher report; sum of four problem scales) 14. There was also some evidence of a bidirectional association. Total difficulties score (parent report) and emotional symptoms (teacher report) at age 4‐5 years were prospectively associated with higher BMI *z* score at 8‐9 years and greater change in BMI *z* score between 4‐5 and 8‐9 years.

A review 4 investigating the relationship between childhood obesity and psychological problems highlights the need for empirical evidence to identify mediating factors that can explain prospective associations between BMI and psychological problems and between psychological problems and later BMI. In children, the stigma associated with having overweight and being teased by peers 4, 5, 15 has been suggested as a potential mediator of the relationship between higher BMI and psychological problems 14, 16. However, little consideration has been given to potential mediators of the reverse association; that is, how early psychological problems may contribute to higher BMI.

Eating behaviors (also referred to as eating “styles” or “traits”) of both children and adults have consistently been associated with energy intake and BMI 17. In particular, “food approach” behaviors such as food responsiveness and emotional overeating are positively associated with higher BMI and excess weight gain 17. As such, these potentially obesogenic eating behaviors may be considered indicators of poor self‐regulation of energy intake. A study of children aged 9 to 12 years (*N* = 292) examined the association between parent‐reported obesogenic eating behaviors (emotional eating, external eating, and restrained eating; Dutch Eating Behaviour Questionnaire 18) and psychological problems 19. All three eating behaviors were significantly associated with more psychological problems (parent‐reported on the Child Behavior Checklist 20). These findings suggest that the relationship between psychological problems and BMI could be mediated by the obesogenic eating behaviors that are typically regarded to be antecedent to excess weight gain.

While there may be indirect support for the importance of eating behaviors in explaining the relationship between psychological problems and BMI in early childhood, most recent large‐scale studies examining child BMI and psychological problems 1, 10, 13, 14 have not collected data on eating behaviors. The current study sought to address this gap by assessing whether psychological problems were associated with higher child BMI *z* score and whether this relationship was mediated by obesogenic eating behaviors in a community sample of 3.5‐ to 4‐year‐olds.

## Methods

### Study design and participants

The present study involved cross‐sectional secondary analysis of data from 194 participants allocated to the control group of the NOURISH randomized controlled trial (RCT) 21, 22. A consecutive sample of first‐time mothers was approached at maternity hospitals in two Australian cities (Adelaide and Brisbane) in 2008 to 2009. Only primiparous mothers 18 years of age or older who had delivered a healthy infant (>35 weeks, ≥ 2500 g), were able to write and speak in English, and who had no documented history of domestic violence or drug or alcohol abuse were eligible. When recontacted at approximately 4 months post partum, 698 mother‐infant dyads were enrolled, representing a 44% consent rate (excluding noncontacts). Compared to nonconsenters, mothers who consented were older (30 vs. 28 years) and more likely to have a university education (58% vs. 36%). Following baseline assessments (infant age: mean = 4.3, standard deviation [SD] = 1.0 months), mother‐infant dyads were allocated to the intervention or control group. Follow‐up assessments as part of the RCT design occurred at infant ages 14 and 24 months. This study used baseline data and cross‐sectional data collected at the long‐term follow‐up when children were aged 3.5 to 4 years.

For the purposes of this study, data were available from 194 of the 346 mothers allocated to the control group. There were no statistically significant differences in terms of maternal age (*P* = 0.62), baseline BMI (*P* = 0.60), or infant birth weight (*P* = 0.88) between those included and those excluded due to missing data. However, a larger proportion of those included had a university education compared to those excluded from the study (66% vs. 47%, *P* < 0.001).

The NOURISH RCT was approved by Human Research Ethics Committees covering Queensland University of Technology, Flinders University, and all the recruitment hospitals and was registered with the Australian and New Zealand Clinical Trials Registry (ACTRN12608000056392).

### Measures

#### Participant characteristics

Maternal and child characteristics (Table 1) were collected at first contact (maternal age, maternal education, child gender). Maternal BMI was calculated based on weight and height measured by trained assessors at baseline (child age 4 months). Duration of breastfeeding (weeks) was based on maternal reports corroborated across all time points (excluding at birth). Age of introduction to solid foods (weeks) was based on maternal report at child age 14 months.

**Table 1:** Characteristics of mother‐child dyads (*N* = 194)

	Mean ± SD or %
***Maternal***	
**Age at delivery (y)**	30 ± 5
**University education**	66
**BMI (kg/m^2^)** a	26 ± 6 (*n* = 193)
***Child***	
**Gender (male)**	52
**Birth weight *z* score**	0.39 ± 0.93 (range = −1.90 to 2.74)
**Age (mo)**	47 ± 3 (range = 41 to 50)
**BMI‐for‐age *z* score** b	0.56 ± 0.86 (range = −1.49 to 4.29)
**Weight status** c	
**Underweight (BMI *z* score < −2)**	0
**Healthy weight (BMI *z* score between −2 and +2)**	97
**Overweight (BMI *z* score > 2)**	3
**Breastfeeding duration (wk)**	35 ± 26 (*n* = 189)
**Age solids introduced (wk)**	23 ± 5 (*n* = 180)

a Measured height and weight at NOURISH baseline (mean infant age = 4.3 ± SD = 1.0 mo) 21.

b Based on measured weight and height and converted to gender‐ and age‐adjusted BMI *z* score using WHO Anthro 26.

c Based on WHO definitions 27.

#### Strengths and Difficulties Questionnaire (SDQ)

The SDQ is a widely used, reliable, and valid 25‐item tool that comprises five scales (five items each). Scales assess children's internalizing (emotional symptoms, peer problems) and externalizing (hyperactivity, conduct problems) problems and prosocial behavior 11, 12, 23. Items are scored 0 (not true), 1 (somewhat true), and 2 (certainly true) and summed for each scale (some items are reverse‐scored). Reliability estimates in the present sample were between 0.50 and 0.75 (Table 2).

**Table 2:** Pearson's correlations between scale scores on the parent‐report SDQ and child BMI *z* score at age 3.5‐4 years (*N* = 194)

SDQ scale (Cronbach's α)	Mean ± SD	Emotional symptoms	Conduct problems	Peer problems	Prosocial behavior	BMI *z* score
**Hyperactivity (0.75)**	4.30 ± 1.38	0.17*	0.41**	−0.04	−0.28**	−0.061
**Emotional symptoms (0.56)**	1.37 ± 1.40		0.30**	0.12	−0.06	−0.04
**Conduct problems (0.56)**	2.21 ± 1.59			0.23**	−0.36**	0.06
**Peer problems (0.50)**	1.49 ± 1.48				−0.31**	0.21**
**Prosocial behavior (0.67)**	7.77 ± 1.71					−0.15*

Range 0‐10, with higher scores indicating more problems/strengths; BMI *z* score based on measured weight and height and converted to gender‐ and age‐adjusted BMI *z* score using WHO Anthro 26.*P < 0.05, **P < 0.01.

SDQ, Strengths and Difficulties Questionnaire.

#### Children's Eating Behaviour Questionnaire (CEBQ)

The CEBQ is a widely used 35‐item tool with demonstrated reliability and validity that measures food approach (food responsiveness, enjoyment of food, emotional overeating, desire to drink) and food avoidance (satiety responsiveness, slowness in eating, emotional undereating, fussiness) eating behaviors 24. Items are scored on a Likert‐style scale from 1 to 5. Mean scores for each scale are calculated (some items were reverse‐scored), with higher mean scores representing a higher expression of that behavior. High overlap between the satiety responsiveness and slowness in eating scales has been consistently reported in the literature 24, 25; thus, these were combined to create a single mean score for satiety responsiveness/slowness in eating. Reliability estimates in the present sample were between 0.74 and 0.92 (Table 3).

**Table 3:** Pearson's correlations between scale scores on the parent‐report CEBQ and child BMI *z* score at age 3.5‐4 years (*N* = 194)

CEBQ scale (Cronbach's α)	Mean ± SD	Fussiness	Emotional undereating	Food responsiveness	Enjoyment of food	Emotional overeating	Desire to drink	BMI *z* score
**Satiety responsiveness/slowness in eating (0.83)**	3.05 ± 0.55	0.50**	0.21**	−0.29**	−0.61**	0.03	−0.02	−0.19*
**Fussiness (0.92)**	1.90 ± 0.81		0.20**	−0.13	−0.66**	0.05	0.07	0.05
**Emotional undereating (0.76)**	3.00 ± 0.81			0.14*	−0.10	0.39**	0.12	0.04
**Food responsiveness (0.75)**	2.41 ± 0.66				0.38**	0.50**	0.33**	0.23**
**Enjoyment of food (0.90)**	3.76 ± 0.70					0.10	0.03	0.04
**Emotional overeating (0.74)**	1.66 ± 0.52						0.15*	0.25**
**Desire to drink (0.83)**	2.73 ± 0.86							−0.04

Mean scores range 1‐5, with higher scores indicating higher level of the eating behavior; BMI *z* score based on measured weight and height and converted to gender‐ and age‐adjusted BMI *z* score using WHO Anthro 26. *P < 0.05, **P < 0.01.

CEBQ, Children's Eating Behavior Questionnaire.

#### Child BMI *z* score

Gender‐ and age‐adjusted child BMI *z* score at 3.5 to 4 years of age was calculated using WHO Anthro software 26 based on weight and height collected by trained study staff using a standardized protocol in which children were measured without footwear or outer clothes using standardized equipment 21. Underweight, healthy, and overweight classifications were made based on World Health Organization (WHO) child growth standards (birth to age 5): underweight, BMI *z* score < −2; healthy weight, BMI *z* score between −2 and +2; and overweight, BMI *z* score > 2 27.

### Data analysis

Statistical analyses were conducted using SPSS^®^ Statistics Version 21 (IBM Corp., Armonk, New York). Parametric bivariate analyses were used to explore the association between potential confounding variables (maternal education, maternal BMI, child gender, birth weight *z* score, breastfeeding duration [weeks], and age first introduced to solids [weeks]) and SDQ and CEBQ mean scale scores and child BMI *z* score. Pearson's correlations were used to assess associations between mean scale scores on the SDQ and CEBQ and between mean scale scores on both tools and child BMI *z* score. Significant associations were followed up, adjusting for selected covariates (in which *P* < 0.10) using multivariable linear regressions.

Mediation analysis was performed only when eating behaviors (mediators) were significantly associated with both the independent variable (SDQ scale) and the outcome variable (BMI *z* score, controlling for the independent variable). To test multiple mediators simultaneously (if more than one obesogenic eating behavior was a potential mediator), the approach outlined by Preacher and Hayes 28 was used. The SPSS PROCESS macro 29 was used to test for specific indirect effects and total indirect effects (combination of all specific indirect effects) as well as the direct effect of the independent variable on the outcome, controlling for the mediators. The significance of indirect and direct effects was assessed using bias‐corrected bootstrap 95% confidence intervals (*n* = 5,000).

## Results

Characteristics of the participants are shown in Table 1. Two‐thirds of mothers held a university‐level degree and were on average 30 years old at the time of their child's birth. Children (52% male) were on average 47 ± 3 months at the time of assessment, and 97% had a BMI *z* score within the healthy weight range.

### Associations between mean scale scores of SDQ, CEBQ, and child BMI *z* score

Pearson's correlations between SDQ scales and child BMI *z* score are shown in Table 2. Peer problems score was positively associated with BMI *z* score. An inverse relationship between prosocial behavior and BMI *z* score was also found. Maternal BMI was the only covariate associated with child BMI *z* score (*P* < 0.001); therefore, multivariable regression analyses were run to confirm whether the associations between these two scales of the SDQ and child BMI *z* score remained significant after adjusting for maternal BMI. In both instances, the associations with BMI *z* score remained significant: peer problems (β = 0.17, *P* = 0.011) and prosocial (β = ‐0.138, *P* = 0.044).

The Pearson's correlations between the CEBQ scales and child BMI *z* score are shown in Table 3. Of relevance to the planned mediation testing were the significant positive associations between obesogenic eating behavior subscales of food responsiveness and emotional overeating with child BMI *z* score. These associations with BMI *z* score remained significant after adjusting for maternal BMI: food responsiveness (β = 0.22, *P* = 0.001) and emotional overeating (β = 0.21, *P* = 0.002).

Pearson's correlations between SDQ and CEBQ scales are shown in Table 4. Emotional symptoms score was associated with greater emotional undereating, emotional overeating, and food responsiveness. Conduct problems score was associated with more emotional undereating and was marginally significantly associated with higher food responsiveness (*P* = 0.044). Peer problems score was associated with higher emotional overeating and food responsiveness. None of the covariates were associated (at *P* < 0.10 level) with emotional undereating or food responsiveness; therefore, no adjustment for covariates was made. However, maternal BMI was associated (*P* = 0.062) with emotional overeating; therefore, adjusted analyses were conducted to confirm the relationship between emotional overeating and emotional symptoms (β = 0.17, *P* = 0.018) and peer problems (β = 0.18, *P* = 0.015).

**Table 4:** Pearson's correlations between scale scores on the parent‐report SDQ and CEBQ at age 3.5‐4 years (*N* = 194)

	CEBQ scale						
SDQ scale	Satiety responsiveness/slowness in eating	Fussiness	Emotional undereating	Food responsiveness	Enjoyment of food	Emotional overeating	Desire to drink
**Hyperactivity**	0.13	0.15*	0.08	0.06	−0.19*	0.06	0.14
**Emotional problems**	0.10	0.10	0.21**	0.26**	−0.01	0.19*	0.09
**Conduct problems**	0.10	0.08	0.20**	0.15*	−0.13	0.13	0.04
**Peer problems**	0.02	−0.06	−0.04	0.16*	−0.01	0.18*	<0.001
**Prosocial behavior**	0.13	−0.04	−0.03	−0.09	0.08	−0.10	0.01

*P < 0.05, **P < 0.01.

SDQ, Strengths and Difficulties Questionnaire; CEBQ, Children's Eating Behavior Questionnaire.

### Mediation analysis

Table 5 shows the tests of specific indirect effects, total indirect effects, and direct effects of the mediation models. Emotional overeating and food responsiveness fully mediated the relationship between emotional problems and BMI *z* score. Overall emotional problems, food responsiveness, and emotional overeating accounted for 8.78% of the variance in BMI *z* score, *F*(3, 190) = 6.10, *P* < 0.0006. In contrast, the relationship between peer problems and BMI *z* score was partially mediated by emotional overeating but not by food responsiveness. Peer problems, food responsiveness, and emotional overeating accounted for 10.10% of the variance in BMI *z* score, *F*(3, 190) = 7.11, *P* = 0.0001. Finally, the relationship between conduct problems and BMI *z* score was fully mediated by food responsiveness; however, conduct problems and food responsiveness accounted for only 5.33% of the variance in BMI *z* score, *F*(2, 191) = 5.38, *P* = 0.0053.

**Table 5:** Direct and indirect effects of SDQ scale scores on BMI *z* score through obesogenic eating behaviors measured via the CEBQ (*N* = 194)

	BMI *z* score	
Mediation model	Bootstrap estimate (SE)	BC 95% CI
**Emotional problems (direct effect)**	−0.070 (0.044)	−0.157 to 0.018
**Emotional overeating (indirect effect)**	0.021 (0.013)*	0.002 to 0.054
**Food responsiveness (indirect effect)**	0.027 (0.015)*	0.005 to 0.065
**Total indirect effect**	0.048 (0.018)*	0.019 to 0.090
		
**Peer problems (direct effect)**	0.094 (0.041)*	0.013 to 0.174
**Emotional overeating (indirect effect)**	0.016 (0.012)*	0.0002 to 0.046
**Food responsiveness (indirect effect)**	0.012 (0.009)	−0.0002 to 0.046
**Total indirect effect**	0.028 (0.013)*	0.008 to 0.059
		
**Conduct problems (direct effect)**	0.013 (0.039)	−0.063 to 0.089
**Food responsiveness (indirect effect)**	0.018 (0.010)*	0.003 to 0.045

BMI *z* score based on measured weight and height and converted to gender‐ and age‐adjusted BMI *z* score using WHO Anthro 26. Unstandardized coefficients reported. *significant effect (CI does not include zero).

CI, confidence interval; BC, bias corrected 95% confidence interval; SDQ, Strengths and Difficulties Questionnaire; CEBQ, Children's Eating Behaviour Questionnaire.

## Discussion

The aim of this paper was to investigate the cross‐sectional associations between children's psychological problems and eating behaviors and to test whether obesogenic eating behaviors mediate the expected positive association between psychological problems and BMI *z* score. The key findings were that both internalizing problems scores (peer pressure and emotional symptoms) were positively associated with obesogenic eating behaviors (food responsiveness and emotional overeating) and peer problems was directly associated with higher BMI *z* score. Multiple mediation models (i.e., simultaneously testing multiple obesogenic eating behaviors as mediators) revealed that (i) emotional overeating and food responsiveness fully mediated the relationship between emotional problems and BMI *z* score, and (ii) emotional overeating, but not food responsiveness, partially mediated the relationship between peer problems and BMI *z* score. Neither of the externalizing problems (hyperactivity and conduct problems) scores were directly associated with BMI *z* score, and hyperactivity was not positively associated with obesogenic eating behaviors. However, conduct problems was positively associated with food responsiveness, which fully mediated the relationship between conduct problems and BMI *z* score. Finally, although prosocial behavior score was weakly inversely associated with BMI *z* score, it was not associated with any eating behaviors; therefore, a mediation model was not tested.

Peer problems score was positively associated with both BMI *z* score and obesogenic eating behaviors (emotional overeating and food responsiveness) in the present sample. However, only emotional overeating partially mediated the association between peer problems and child BMI *z* score. It may be that for children who are experiencing problems interacting with peers, food is used as a source of emotional comfort. However, due to the cross‐sectional nature of the study, the possibility of reverse causality remains. Jansen and colleagues 1 found evidence for a bidirectional effect between peer problems score and BMI *z* score. However, when children with overweight at baseline (age 4‐5 years) were excluded from the analyses, the relationship between peer problems predicting BMI *z* score became nonsignificant, whereas the opposite effect remained 1. Cognizant of the design limitations of the current study, the findings of significant associations between peer problems and two key obesogenic eating behaviors are still informative in terms of the ways in which children who are experiencing problems with peer relationships may also display eating behaviors that may put them at heightened risk of overconsumption and excess weight gain.

Emotional symptoms, conduct problems, and hyperactivity scores were not directly associated with child BMI *z* score in the present study. However, both emotional symptoms and conduct problems were positively associated with obesogenic eating behavior: both were associated with higher food responsiveness. Emotional symptoms were also associated with higher emotional overeating. Food responsiveness and emotional overeating fully mediated the relationship between emotional symptoms and BMI *z* score, and food responsiveness (tested alone) fully mediated the relationship between conduct problems and BMI *z* score. The absence of direct effects with BMI *z* score is perhaps not surprising. No association between emotional symptoms score and BMI *z* score was found at ages 4‐5 or 8‐9 in the LSAC sample. Interestingly, Jansen and colleagues 1 found that a cross‐sectional positive relationship between emotional symptoms and child BMI *z* score did not emerge until 10 to 11 years of age. In terms of conduct problems and BMI, an association was only found in boys in both the LSAC 13 and Millennium 10 cohorts, and hyperactivity has not previously been associated with higher BMI in these cohorts 1, 10, 13, 14. It is also worth noting that emotional symptoms and conduct problems were also positively associated with emotional undereating in the present sample. Emotional under‐ and overeating scales were positively correlated with one another (*r* = 0.39, *P* < 0.01), but only emotional overeating was associated with BMI *z* score. Nevertheless, the positive associations between these two SDQ scales and emotional undereating raises the question of whether the tendency to emotionally undereat may obscure or suppress a direct relationship between emotional symptoms/conduct problems and BMI *z* score. Further exploration of the long‐term impacts of these problems on BMI, in boys and girls separately, seems warranted.

Prosocial behaviors score was related to lower BMI *z* score but was not associated with any of the eating behaviors. No association was observed between prosocial score and BMI in the LSAC sample 1, 13, 14 and was different for boys and girls in the Millennium Cohort Study, in which at age 3 years boys with overweight (but not obesity) had lower prosocial scores relative to normal‐weight boys, whereas girls with obesity had higher prosocial scores relative to normal‐weight girls. Taken together, there is inconsistent evidence regarding the association between prosocial behavior and BMI, and the present study provides no evidence to suggest eating behaviors play a mediating role in the association observed here.

Consistent with the literature, the current findings suggest that psychological or weight‐management programs/interventions for children should consider whether there are comorbid eating and weight‐related concerns. While this study has explored the pathway between psychological problems and BMI by examining eating behaviors as potential mediators, further research is needed to understand the nature of the relationship between psychological problems and eating behaviors. For instance, it may be that obesogenic eating behaviors are a coping mechanism for psychological problems; alternatively, psychological problems that emerge during childhood may reflect an underlying difficult temperament that also manifests as problematic eating behaviors. Past research suggests that temperament is a strong predictor of emotional and behavioral problems in children 30, and difficult temperament has been found to be associated with problematic eating behaviors such as emotional over/undereating 31. A recent study showed an association between surgent temperament (characterized by high impulsivity, activity level, and novelty/pleasure seeking) and high food responsiveness, enjoyment of food, and higher BMI in a sample of low‐income preschoolers. Thus, temperament will be an important variable to also consider in future studies examining psychological problems and BMI.

The use of well‐established and previously validated tools to measure the key variables of interest and the use of measured weight and height data are strengths of this study. However, parent‐report of child behaviors may be subject to bias, and while the CEBQ subscales showed high internal consistency (α values between 0.75‐0.92), the reliability estimates of the subscales of the SDQ were generally modest (α values between 0.50‐0.75). Limitations include the small sample size, which increases the potential for type II error and precluded testing for effect moderators such as gender, socioeconomic status, and ethnicity 4. The effect sizes reported here were small and variance in BMI *z* score explained by the models was low (≥10%), which may in part reflect the small sample size but may also be explained by only three of the children in the present sample having a BMI *z* score above the healthy weight range. Previous studies examining the relationship between the SDQ scales and child BMI have stratified by gender and found differences between boys and girls 10, 13. Therefore, the extent to which the current findings can be directly compared with previous findings is limited because it was not feasible to examine gender as a moderator. The sample was also not representative of the general population, thus limiting the generalizability of the results. Finally, it was not possible to assess causality due to the cross‐sectional design, and the potential for reverse causality must be considered. Future research is needed to examine whether the proposed mediation models can be supported in heavier samples and in longitudinal studies.

## Conclusion

This study is the first to demonstrate the potential for children's obesogenic eating behaviors to mediate a relationship between psychological problems and weight. Almost all children were defined as a healthy weight, which may have attenuated the strength of associations between psychological problems and weight and may limit the generalizability of the results. Future research in this area that can utilize prospective study designs with large representative samples should include measures of potential mediators of these relationships to better understand the complex interplay between weight and psychological well‐being in the context of development.
